# Genome Number and Size Polymorphism in Zika Virus Infectious Units

**DOI:** 10.1128/JVI.00787-20

**Published:** 2021-02-24

**Authors:** Nicole R. Sexton, Eric D. Bellis, Reyes A. Murrieta, Mark Cole Spangler, Parker J. Cline, James Weger-Lucarelli, Gregory D. Ebel

**Affiliations:** a Department of Microbiology, Immunology and Pathology, College of Veterinary Medicine and Biomedical Sciences, Colorado State University, Fort Collins, Colorado, USA; b Department of Biomedical Sciences and Pathobiology, Virginia-Maryland College of Veterinary Medicine, Virginia Tech, Blacksburg, Virginia, USA; University of Texas Southwestern Medical Center

**Keywords:** ZIKV, Zika virus, flavivirus, specific infectivity, barcoded virus, aggregates

## Abstract

Zika virus (ZIKV; *Flaviviridae*, *Flavivirus*) is an arthropod-borne infection that can result in severe outcomes, particularly in fetuses infected *in utero*. It has been assumed that infection by ZIKV, as well as other viruses, is largely initiated by individual virus particles binding to and entering a cell. However, recent studies have demonstrated that multiple virus particles are frequently delivered to a cell simultaneously and that this collective particle delivery enhances infection. ZIKV is maintained in nature between Aedes aegypti mosquitos and vertebrate hosts, including humans. Human infection is initiated through the injection of a relatively small initial inoculum comprised of a genetically complex virus population. Since most mutations decrease virus fitness, collective particle transmission could benefit ZIKV and other arthropod-borne diseases by facilitating the maintenance of genetic complexity and adaptability during infection or through other mechanisms. Therefore, we utilized a barcoded ZIKV to quantify the number of virus genomes that initiate a plaque. We found that individual plaques contain a mean of 10 infecting viral genomes (range, 1 to 212). Few plaques contained more than two dominant genomes. To determine whether multigenome infectious units consist of collectively transmitting virions, infectious units of ZIKV were then separated mechanically by centrifugation, and heavier fractions were found to contain more genomes per plaque-forming unit, with larger diameters. Finally, larger/heavier infectious units reformed after removal. These data suggest that ZIKV populations consist of a variety of infectious unit sizes, likely mostly made up of aggregates, and only rarely begin with a single virus genome.

**IMPORTANCE** The arthropod-borne Zika virus (ZIKV) infects humans and can cause severe neurological sequelae, particularly in fetuses infected *in utero*. How this virus has been able to spread across vast geological ranges and evolve in new host populations is not yet understood. This research demonstrates a novel mechanism of ZIKV transmission through multigenome aggregates, providing insight into ZIKV evolution, immunologic evasion, and better future therapeutic design. This study shows that ZIKV plaques result from collections of genomes rather than individual genomes, increasing the potential for interactions between ZIKV genotypes.

## Abstract (precis)

The arthropod-borne Zika virus (ZIKV) infects humans and can cause severe neurological sequelae, particularly in fetuses infected *in utero*. How this virus has been able to spread across vast geological ranges and evolve in new host populations is not yet understood.

## INTRODUCTION

Arboviruses cause extensive morbidity and mortality in humans, wildlife, and agricultural animals throughout the world (1–3). In addition, many arboviruses such as Zika, chikungunya, dengue, Japanese encephalitis, and Rift Valley fever viruses have emerged in new host species or geographical locations, demonstrating their ability to adapt to new environments and hosts (4, 5). The first human Zika virus (ZIKV) infection was identified by serology in 1952; however, ZIKV was largely ignored until it began spreading across the Pacific Ocean in 2007. Interest peaked with the outbreak in Brazil from 2014 to 2015, which correlated with increased microcephaly among infants born to mothers infected with ZIKV while pregnant (6, 7). Spread into immunologically naive populations linked ZIKV infection to various neurological sequelae and ocular disease among both adults and fetuses (6, 8–10). As of 2 February 2017, the World Health Organization (WHO) reported 2,656 ZIKV-associated microcephaly and/or central nervous system malformation cases across 30 countries and territories. Although this is the last WHO report on neurologic complications, the Centers for Disease Control and Prevention recorded 80 cases of ZIKV across the United States and its territories in 2019 as of 9 January 2020, demonstrating that ZIKV remains a persistent health threat.

RNA viruses exist within the host as genetically diverse mixtures of variants—often called mutant clouds—that individually differ from the consensus nucleotide sequence (11–18). These mutant cloud populations are generated due to replication by error-prone, proofreading-deficient RNA-dependent RNA polymerases (19–24). The generation of a large number of mutations, generally estimated to be one mutation per genome generated, allows for the potential to explore all beneficial mutations one nucleotide change away from the originating genome (21, 25). However, the majority are deleterious (11, 17, 26–28). In fact, many of the viral progeny released from a cell are likely to be unable to replicate at all, and this is supported by high particle/PFU ratios for many viruses (29). To maintain transmission, ZIKV must cyclically infect mosquitos and vertebrate hosts, species separated by hundreds of millions of years of evolution (30). Arbovirus inoculation from a mosquito occurs through the injection of a small volume of saliva containing highly diverse populations of viruses (18, 31–33). ZIKV entry occurs mainly through clathrin-mediated endocytosis, but utilizes a wide variety of receptors, including but not limited to C-type lectins, TIM, and TAM family PS receptors (34). It is unknown whether the most relevant infectious unit for ZIKV is individual particles or whether ZIKV utilizes mechanisms for delivering viral collections, which could enhance the successful delivery of replication competent genomes and shield viruses from neutralization or detection by host immune systems. Recent evidence suggests that many viruses may deliver multiple virions to a single cell, which could allow viruses to compensate for a large population of defective genomes (35–41). This may be particularly important for arboviruses because they must replicate in widely divergent hosts (e.g., mosquitoes and humans), and viral fitness determinants may differ between these hosts. Compensation can occur by complementation, recombination, cooperation, or increasing the probability of a replication-competent virus entering a cell (15, 16, 42). Viruses have been shown to coinfect a cell through direct cell-to-cell spread, polyploidy, aggregations of particles, bacterial delivery of multiple virions, or vesicle packaging of multiple particles (36–43). The number of particles delivered simultaneously has been recorded to range from 2 to more than 15 depending on the virus and the mechanism of delivery (37–39, 41). However, this is likely an underestimate due to limits of detection for assays used. Further supporting the potential delivery of more viruses than has been detected is the observation of up to 21 viruses in a single electron microscopic slice within a vesicle (41, 44). For viruses packaged into vesicles, vesicles are often enriched in phosphatidylserine (PS) (39, 41). Interestingly, PS has been shown to be important in ZIKV entry into cells, although this has been ascribed to binding immature particles (34, 45, 46). Vesicles are generated from the secretory autophagy pathway (40, 47, 48). Autophagy is stimulated by ZIKV infection, and inhibition of autophagy has been linked to decreased replication and severity of ZIKV, whereas induction of autophagy increased the RNA copy number, suggesting a positive role of autophagy on ZIKV replication (40, 49–51). Multiple mechanisms have now been identified that allow viruses to transmit from one cell to another as groups of viruses rather than as individual particles (referred to here as collective particle transmission); ZIKV uses similar cellular pathways during infection. Therefore, we sought here to determine the extent to which multiple genomes initiate plaques, conventionally defined as individual infectious units, for ZIKV using “barcoded” ZIKV and mechanical separation of ZIKV populations.

Our results demonstrate that ZIKV plaques are initiated by an average of 10 genomes. However, in most instances only one or two of these genomes become dominant in the population. ZIKV infectious units can be mechanically separated into fractions of different centrifugal weights, with the heaviest fractions containing more genomes per PFU and having the largest diameters. In addition, we demonstrate that heavier fractions are regenerated, or persist, after attempted removal by centrifugation. Overall, these data suggest that ZIKV infections begin with viruses that are often transmitted collectively, most significantly through viral aggregates. Understanding ZIKV transmission mechanisms and determining the most relevant infectious unit will inform the development and potential pitfalls of treatments and facilitate our understanding of how ZIKV and other arboviruses evolve.

## RESULTS

### Generation and characterization of a high-diversity barcoded ZIKV infectious clone.

**FIG 1:**
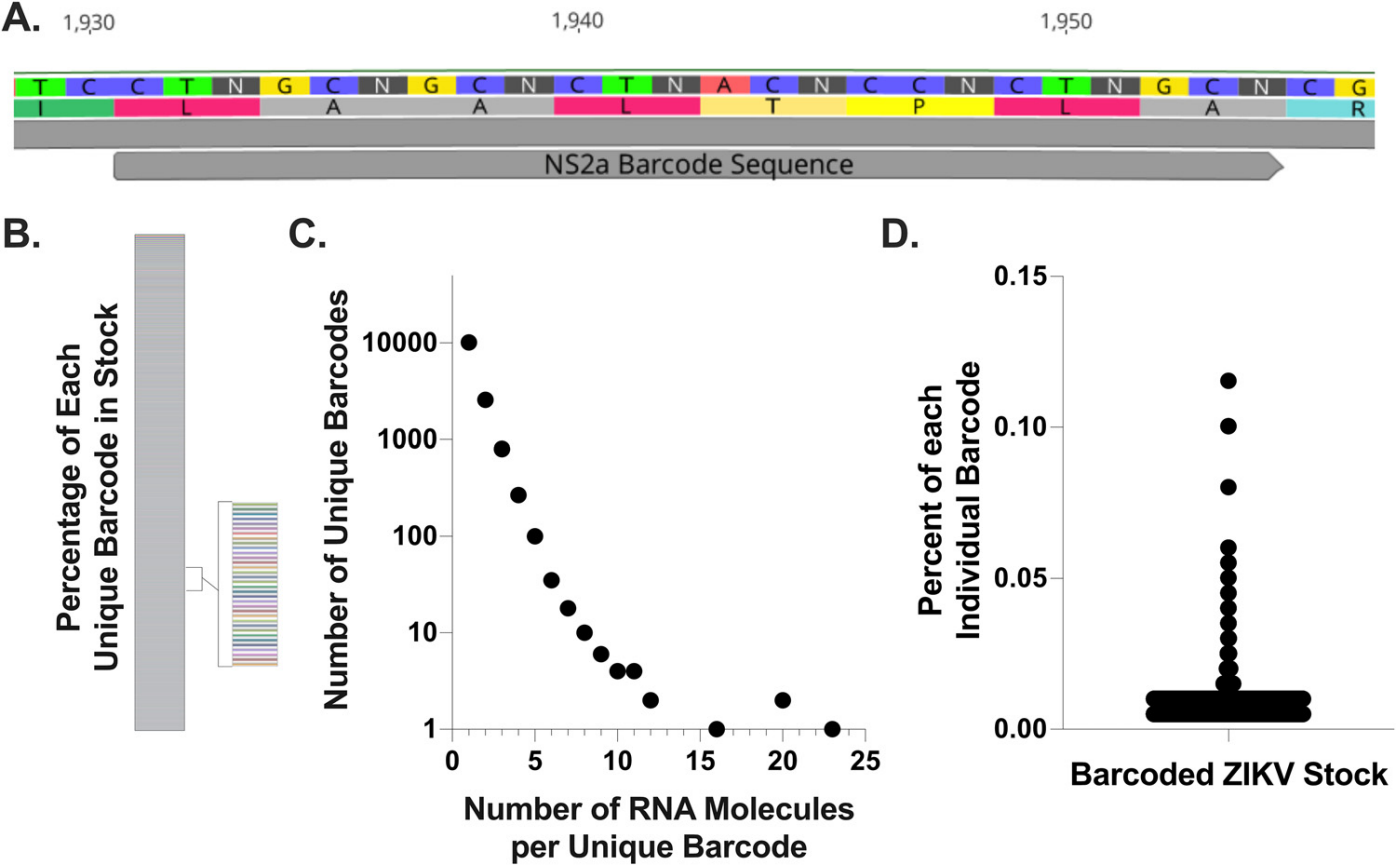
Barcode ZIKV contains high diversity at the engineered degenerate sites. (A) ZIKV was engineered with degenerative nucleotides in the third codon position of an 8-amino-acid stretch of NS2a, without altering the protein. (B to D) Barcode diversity is shown as individual barcodes by the percentage of the whole (B), the number of RNA molecules in the sequenced sample for each barcode identified (C), and the percentage of the total stock made up of each barcode (D).

In order to investigate how many ZIKV genomes initiate a single-cell infection, a ZIKV infectious clone (PRVABC59) was engineered to encode an 8-nucleotide barcode across eight codons within the NS2A protein. This barcode was designed with degenerate nucleotides engineered at the third codon position within an 8-amino-acid stretch of NS2A where identical amino acids are encoded regardless of the identity of the wobble base; thus, the resulting proteins should be identical (Fig. 1A). The theoretical diversity of bar-ZIKV at the barcoded region is 4^8^—or 65,536 uniquely barcoded genomes. A first generation of this barcoded ZIKV (bar-ZIKV), along with barcodes introduced in alternative genetic positions, was previously described and shown not to affect replication kinetics; however, the diversity was far below the theoretical value (52, 53). Here, a second-generation bar-ZIKV was utilized with enhanced diversity in the barcoded region. The second-generations bar-ZIKV virus was generated by transfection in 293T cells instead of Vero cells, allowing efficient delivery of the clone and preventing selection due to multiple rounds of replication, since ZIKV can replicate in but not enter 293T cells. To determine whether this second-generation bar-ZIKV resulted in increased diversity across the barcoded region, a sample of the stock virus was prepared for Illumina sequencing. Using PrimerID technology (54) sequencing reads were collapsed into number of RNA molecules present in the original stock sample per barcode. This bar-ZIKV stock displays up to 13,966 different barcodes in a single Illumina sequencing sample (Fig. 1B and C), with none dominating the population (Fig. 1C and D). The maximum number of RNA molecules representing an individual barcode was 23, with nearly every barcode present represented by a single RNA molecule (Fig. 1C). In fact, the most highly represented barcode comprised only 0.12% of the total population and the majority of individual barcodes comprised less than 0.01% of the population (mean, 0.007%; standard error of the mean [SEM], ±3.95e–005) (Fig. 1D). In addition, only 0.21% of barcode sequences from the bar-ZIKV stock included mutations in the first or second codon position, demonstrating that nearly all barcodes are the result of engineered degeneracy rather than mutations arising during viral recovery. Together, these findings demonstrate that the bar-ZIKV stock is exceptionally diverse and tractable. In addition, they suggest that the diversity of bar-ZIKV at the engineered barcode approaches the theoretical maximum and that it is an appropriate tool to investigate the number of genomes that initiate an infection.

### ZIKV plaques are comprised of a range of infectious unit sizes, but commonly only one genome dominates the resulting viral population.

**FIG 2:**
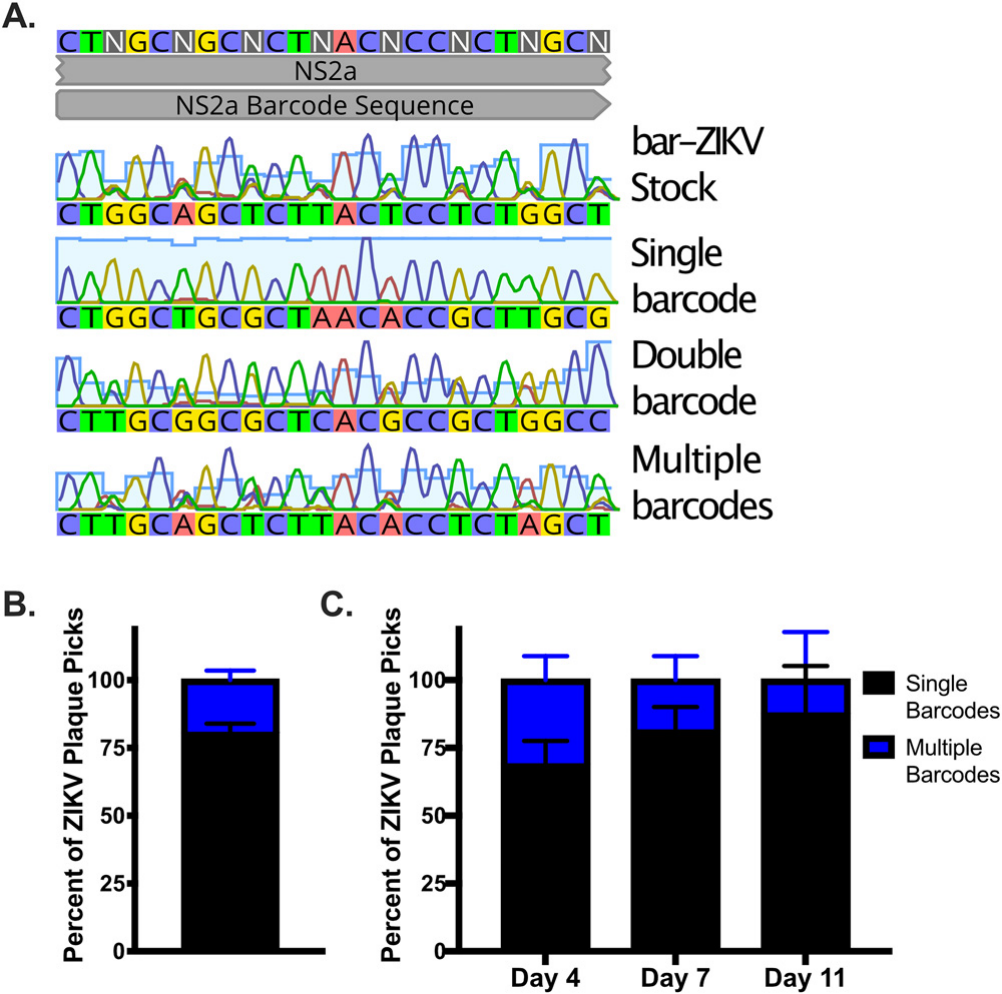
Single and multiple majority barcodes are identified in plaques from bar-ZIKV viral stock, as well as bar-ZIKV-infected mosquito samples. Barcoded ZIKV-infected plaques (Vero cells) were isolated and sent for sequencing across the barcode. (A) Representative chromatograms from plaque purified barcoded ZIKV samples shown. (B and C) Percentages of plaques containing single versus multiple barcode sequences from stock barcoded ZIKV (150 plaques, two independent experiments) (B) or legs/wings from *A. aegypti* mosquitoes injected with barcoded ZIKV at 4, 7, or 11 dpi presented (47 plaques, two independent experiments) (C). Bars in panels B and C indicate the SD.

Having established that bar-ZIKV is a high-diversity tool capable of tracking individual viral genomes, bar-ZIKV stock virus was used to infect African green monkey kidney cells (Vero) by agar overlay plaque assay. Individual plaques were picked and sequenced by Sanger sequencing. Importantly, plaques were picked that were well separated from each other in wells, with from 1 to ∼100 plaques present. Sanger sequencing chromatographs were analyzed across the barcoded region of NS2a for single versus mixed peaks at the third codon positions. Three patterns were observed with ZIKV plaques consisting of (i) only single peaks across the barcode (one majority barcode), (ii) equally mixed double peaks at the third position (suggestive of two majority barcodes), and (iii) highly mixed peaks at the third codon position (multiple majority barcodes) (Fig. 2A). As determined by Sanger sequencing, 80% of ZIKV plaques consisted of one majority barcode, with 20% consisting of more than one majority barcode (Fig. 2B). The multiple barcodes could further be divided into ∼19% likely double barcodes and ∼1% multiple barcodes of unknown number. To determine whether this distribution is also present in a more natural infection course, *A. aegypti* mosquitoes were intrathoracically injected with bar-ZIKV, and legs and wings collected at days 4, 7, and 11 postinjection. Leg and wing samples were not homogenized but instead allowed to perfuse in mosquito dilutant in order to minimize disruption of any collective viral particles present. A similar distribution was observed from *A. aegypti* legs and wings with ∼69% single barcodes and ∼31% multiple barcodes on day 4 (Fig. 2C). As would be expected as a result of bottlenecks in barcodes during serial cell infections within mosquitoes, the percentage of mixed barcode plaques decreased over time, with only ∼81% single barcodes and ∼19% multiple barcodes on day 7 and ∼88% single barcodes and ∼12% multiple barcodes on day 11 postinjection (Fig. 2C).

**FIG 3:**
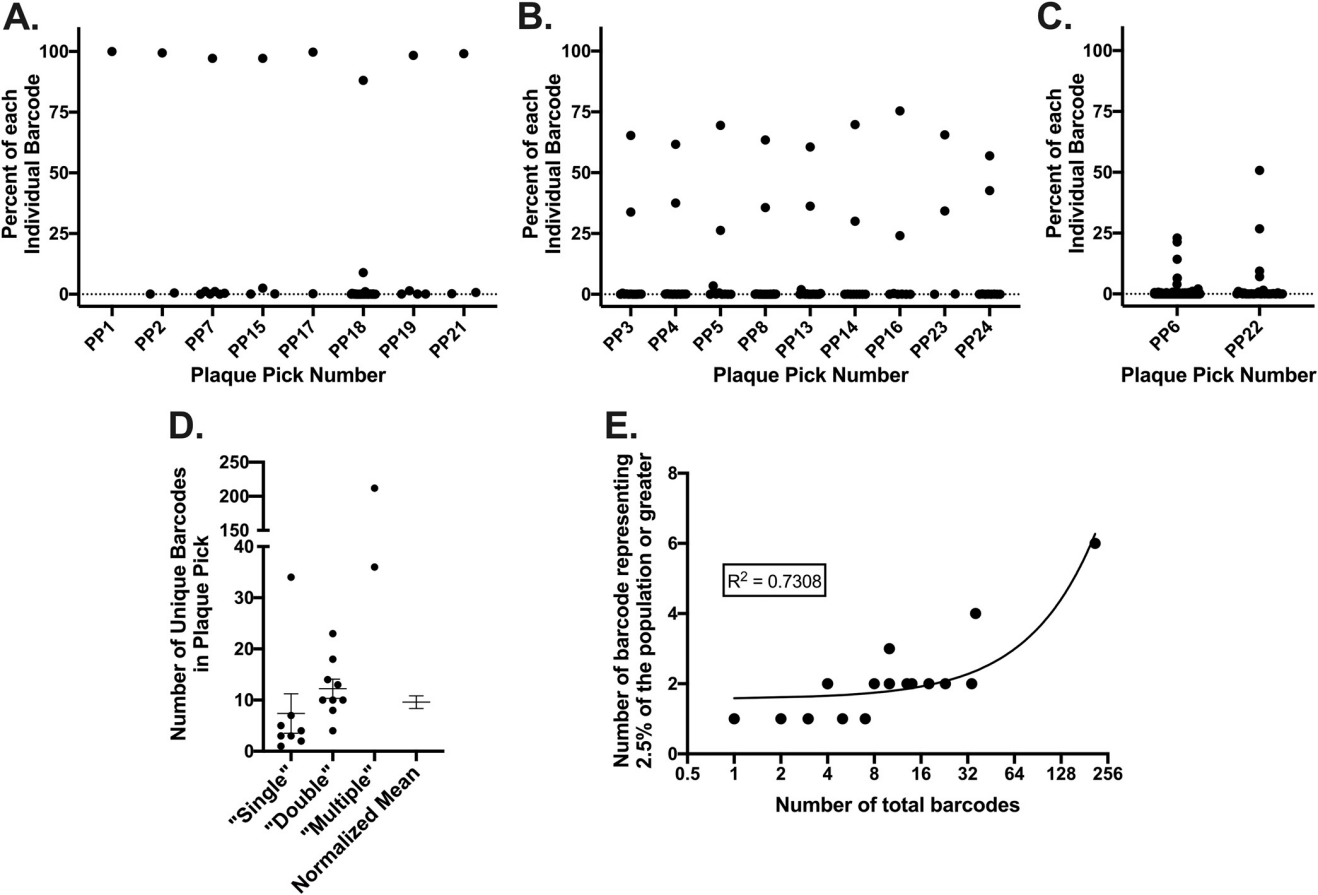
The number of genomes contained in a plaque is rarely one. A subset of plaque isolated samples Sanger sequenced were selected to include majority single, double, and multiple barcode-containing plaques. RNA was transcribed to cDNA using PrimerID tags to bioinformatically identify individual RNA molecules. Illumina sequencing was performed across the barcoded region. Individual barcodes are graphed as a percentage of the total barcode population per plaque for single (A), double (B), and multiple (C) majority barcode plaques. (D) The number of unique barcodes per plaque is graphed for each majority group, as is a normalized mean across all plaques. (E) The number of barcodes present at 2.5% of the population or higher is graphed as a function of the total barcodes in each plaque (*R*^2^ = Pearson’s correlation coefficient). Barcodes identified were excluded if they were represented by only one RNA molecule, if they contained alternative nucleotides in the first or second codon position within the barcode region, or if they were a minority barcode one mutation away from a well-represented barcode (>50 RNA copies).

Since Sanger sequencing can only detect majority nucleotides and cannot distinguish between linked and unlinked nucleotides, a subset of the samples was sequenced using the Illumina HiSeq4000 platform. Similar to the stock bar-ZIKV virus, samples were prepared with the addition of PrimerID tags allowing for postsequencing bioinformatic condensing of individual RNA molecules. Samples were chosen to represent the Sanger sequencing groups of single, double, and multiple predicted barcodes. All major barcodes were consistent between Sanger and Illumina sequencing (data not shown), and those predicted to contain single, double, or multiple majority barcodes determined by Sanger sequencing contained only one, two, or many highly represented barcodes by Illumina, respectively, consistent with the Sanger sequencing results (Fig. 3A to C). Overall, the Illumina sequencing results supported the Sanger sequencing results in demonstrating that in most plaques only a single ZIKV genotype becomes dominant in the plaque population with a subset having two dominant genotypes and a minority consisting of mixed competitive genotype populations within a single plaque.

After bioinformatically reducing the sequencing results to individual RNA molecules, data were further refined by removing all barcodes with mutations present outside the third barcode position, though very few existed. In addition, all barcodes represented by only one RNA molecule were removed from the data set, as were all barcodes only one mutation away from a majority, or well-represented (>50 RNA molecules), barcode. Together, this removed barcodes that were likely to have been generated through mutations during replication within the plaque rather than barcodes initially introduced to the plaque during infection. Sequencing results used for further analysis therefore had identical first and second codon positions with highly variable third codon positions indicative of an engineered barcode and were condensed from many sequencing reads into individual RNA molecules containing specific barcodes (Table 1). Even after these stringent cutoffs for barcode inclusion, only one plaque resulted in the presence of a single barcode by Illumina sequencing; all others included a low-level presence of additional barcode populations (Table 1 and Fig. 3A to E). Within the group of plaques with single majority barcodes there were an average of 7.375 (± 3.859 SEM) barcodes present (range, 1 to 34), within the two major barcodes set there were 12.22 (± 1.877 SEM) barcodes (range, 4 to 23), and the mixed majority barcodes plaques sequenced had either 36 or 212 barcodes detected. The combined mean when corrected for the percentage of the total plaques each group made up was 9.6 (± 1.227 SEM) barcodes per plaque (Fig. 3D). Since the average number of total barcodes per plaque increased with the number of dominant barcodes per plaque, the data were also analyzed as the number of barcodes representing 2.5% of the population or more in an individual plaque as a function of the total number of barcodes in that plaque (Fig. 3E). A linear association between the total number of barcodes initiating a plaque and the number of barcodes that are replication competitive in the viral population within a plaque was identified (*R*^2^ = 0.7308, linear regression). These data demonstrate that the number of genomes present in an individual plaque varies considerably and are rarely made up of individual starting genomes. Further, these data suggest that replication-competent/competitive genomes may make up only a subset of delivered genomes (∼1:9).

### ZIKV infectious units can be separated into light, medium, and heavy fractions, where the heavy fraction contains significantly more genome equivalents and larger particles.

**FIG 4:**
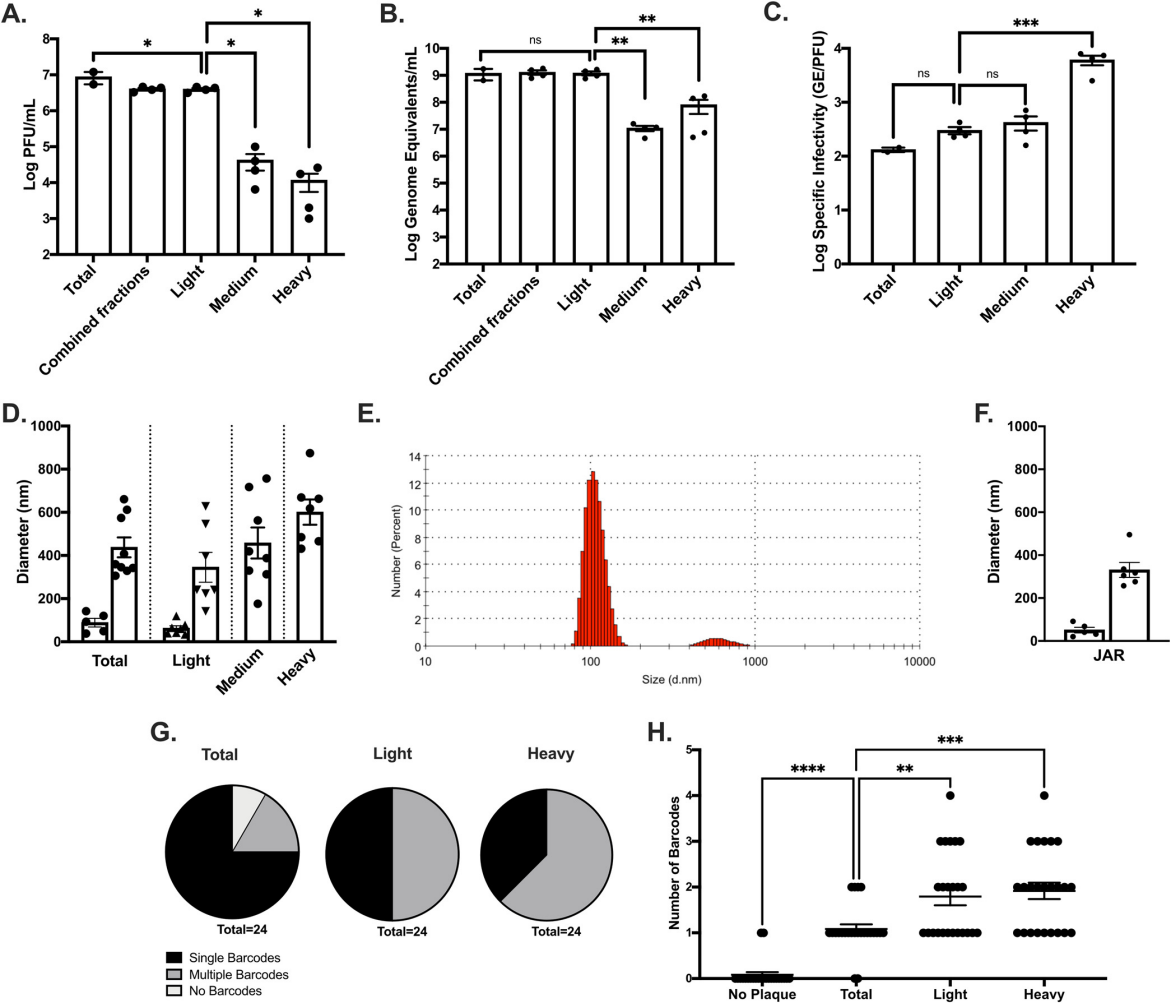
ZIKV infectious units can be separated into light and heavy fractions with different particle diameters. Vero cells were infected with a ZIKV infectious clone at an MOI (PFU/cell) of 1 in 10% FBS media (A to C, G and H) or serum-free media (D to F). At 2 dpi (5 dpi for JAR), the supernatants were collected and clarified. (A to D, G and H) Particles in supernatant were separated into three fractions: light (predicted ≤100-nm particle size), medium (predicted 100- to 200-nm particle size), and heavy (predicted ≥200-nm particle size). Washes were performed to remove 50- or 100-nm carryover. Plaque assays were performed to determine fraction titers (A), RT-qPCR from identical samples was performed to determine the genome equivalents (GE) (B), and the specific infectivity was determined (GE/PFU) (C). (D to F) Total or fractionated supernatants were similarly prepared for DLS fixed in 2.5% glutaraldehyde and diluted in PBS. Samples were read on a Zetasizer Nano ZS. (E) Total supernatant from Vero cells is shown as the number of particles in each size group by DLS. (F) DLS measurements were taken for total supernatants generated in JAR (human placental) cells. (G and H) Single- versus multiple-barcode-containing plaques, as determined by using Illumina MiSeq (ultralow variants not present due to a lower read depth). Error bars represent the SEM; comparisons were determined by one-way ANOVA (*, *P* < 0.05; **, *P* < 0.005; ***, *P* < 0.0005; ****, *P* < 0.0001).

The observation of an average of 10 (and up to 212) uniquely introduced barcodes within individual plaques suggested that ZIKV infectious units exist that are significantly larger, and thus heavier, than individual particles. Individual ZIKV virions are 50 nm in diameter (34); theoretically, 6 virions could be aggregated or packaged together with a diameter of ∼100 nm, and an extracellular vesicle with a diameter of 350 nm (typical of a microvesicle [41, 55]) could contain an average of 220 ZIKV particles, assuming random packaging. To test whether infectious units exist that are significantly larger than individual virions, Vero cells were infected with a ZIKV infectious clone (PRVABC59) at a multiplicity of infection (MOI) of 1, and supernatants were collected at 2 days postinfection (dpi). Cellular debris was removed by pelleting, then ZIKV samples were mechanically separated by centrifugation into three fractions: light, medium, and heavy (predicted particle sizes ≤100 nm, 100 to 200 nm, and ≥200 nm, respectively, in diameter). Pellets were washed five times to remove small particle carryover. Washes were predicted to remove greater than 99% of 50- or 100-nm-diameter particle carryover in the medium and heavy fractions, respectively. It is important to note that these diameter cutoffs are only accurate for spherical vesicles with the predicted density (1.17 g/cm^3^ based on WNV virions [56]). With aggregates or vesicles of different density, such as linearly bound rather than densely packed aggregates, other factors such as buoyancy would alter what size particles are pelleted (57). Immediately after fractionating, ZIKV light-, medium-, and heavy-fraction samples were titrated by plaque assay to determine whether infectious units were present. The majority of PFU were found in the light fraction; however, the medium and heavy fractions also contained ZIKV infectious units (Fig. 4A). RNA was extracted from the same samples and tested for the number of genome equivalents (GE) present by reverse transcription-quantitative PCR (RT-qPCR); again, ZIKV RNA was found in all fractions (Fig. 4B). Since viral particles could be heavy due to factors other than multiple viral particles aggregating or being packaged together, we next assessed specific infectivity (GE/PFU) of each fraction. There was no difference in the specific infectivity between total (unfractionated ZIKV), light, or medium fractions. However, the ZIKV heavy fraction contained significantly more GE per PFU compared to the other groups (one-way analysis of variance [ANOVA], *P* = 0.0004) (Fig. 4C). These data support the hypothesis that ZIKV infectious units are made up of mixed populations including aggregates or other collective particles of variable size.

To better determine the sizes of aggregates found in the different ZIKV infectious unit fractions we next measured particle sizes by dynamic light scattering (DLS). DLS uses the Brownian motion of particles to infer particle size (58). Importantly, DLS measurements only provide measurements of the longest diameter for particles or aggregates. Thus, in the case of ZIKV particles, individual virion should measure near 50 nm (34). However, aggregates of two to six particles, if tightly packaged, could all measure at ∼100 nm. If aggregates are instead lined up in rows, then six particles would measure at ∼300 nm. ZIKV samples were prepared similarly to those used to determine titers and GE, except that infections were performed in serum-free media in order to prevent measurement of particles present in fetal bovine serum (FBS). Total supernatants, as well as each fraction (light, medium, and heavy), were fixed with 2.5% glutaraldehyde as soon as they were prepared to preserve aggregates. In addition to ZIKV-infected samples, conditioned medium incubated with Vero cells for 2 days was also fixed and diluted, and particles were measured by DLS. Conditioned medium did not result in quality DLS measurement, as would be expected, and the only consistent particles identified had <1-nm diameters. Some extremely large particles were occasionally observed (∼5,500-nm diameter) as is typical from dust or other contaminants, but these were minor, rare, and random components. Of note, no particles were observed in media that were detectable when converted for percent volume in the media, demonstrating their rarity in the solvent (data not shown). Measurements from the conditioned media demonstrated that any particles found as a significant portion of the media in ZIKV-infected supernatant with diameters of 50 nm or greater are a result of ZIKV infection rather than normal cell processes, although they could result from cell damage as a result of infection rather than viral particles.

Having demonstrated that Vero cells do not generate ZIKV-like particle sizes, we next measured total ZIKV samples. All ZIKV samples provided good DLS measurements with clean correlogram and cumulant fit data (data not shown). The particles <1 nm in diameter observed in conditioned media were observed in some ZIKV samples but were a minor part of the particle population and often were not observed at all. Instead, ZIKV total supernatants were made up of two major particle populations: one small particle group averaging 76 nm (typically either ∼50- or ∼120-nm diameter particles were observed), and one larger particle group averaging 422 nm (Fig. 4D). In order to understand what percentage of the ZIKV particle population belonged to each particle size group, DLS data were analyzed as the number of particles predicted to be in each group. In total ZIKV supernatants, the majority of particles are in the smaller particle group rather than in the larger group, consistent with the majority of PFU being present in the light fraction (Fig. 4E). In order to better understand what particle sizes make up light, medium, and heavy ZIKV fractions, these samples were also measured by DLS. Similar to the total ZIKV population, the light fraction contained two particle size populations with the small particles averaging 67-nm diameters and the larger particles averaging 352-nm diameters. In contrast, the medium and heavy fractions only had single populations of particle sizes of 517 and 706 nm, respectively (Fig. 4D). Together, these data show that particle sizes increase between the light, medium, and heavy fractions of ZIKV-infected supernatants from Vero cells and that these particles are stimulated directly by infection with ZIKV.

To determine whether particle size groups were specific to Vero cells or also present in ZIKV populations generated from other cell types, a human placental cell line (JAR cells) was similarly infected with the ZIKV infectious clone. Similar particle groups were observed after replication in JAR cells with small particles averaging 55-nm diameters and the large particles averaging 342-nm diameters (Fig. 4F). It is important to note that, in contrast to Vero cells, JAR conditioned media alone provide quality DLS data with particle sizes of below 1 nm in diameter, as well as a second repeatable population in the 200-nm diameter range. The larger particle sizes were a small portion of the total particles present since they were not observable when analyzed by volume instead of intensity measurements and they are smaller than the large particles observed in JAR ZIKV-infected supernatants. However, this does demonstrate that JARs are prone to generating a consistent particle in the same size range as potential ZIKV aggregates or collectively packaged virions in the absence of infection, making controlling for particles generated by this cell type important. Still, ZIKV-infected JAR cells do demonstrate ZIKV-specific particle generation, and particles in the size range of individual and aggregate ZIKV particles are significantly more abundant in JAR media after infection, suggesting that a distribution of free and aggregated ZIKV virions occurs in different cell types.

### Diversity of barcodes between light and heavy ZIKV fractions demonstrates a nonlinear division of genotypes.

We next sought to determine whether there were differences between the number of genomes present in a plaque from the fractionated ZIKV populations. Total, light, and heavy fractions were prepared and immediately used to infect Vero cells as previously described. When plaques were visible, well-separated plaques were isolated, and samples were prepared for sequencing on the Illumina MiSeq platform. In order to control for mixed plaques resulting from infections that are not visible by eye or residual genomes from uninfectious viral particles, matched plaque-free areas of the cell monolayer were isolated and also prepared for sequencing. The sequencing depth for these samples was lower than the previous plaque pick data sequenced on the HiSeq platform, so extreme minority variants were not observable, making these data most comparable to Fig. 3E and Sanger sequencing (Fig. 2). Single barcodes in the total supernatant made up 75% of plaques, multiple barcodes represented 16.7% of plaques, and 8.3% of plaques had no barcodes (Fig. 4G). RNA isolated from the cell monolayer without visible plaques resulted in no barcodes identified in 22 of 24 samples (91.7%), but two samples resulted in either two or five RNA molecules of a single barcode represented (Fig. 4H). This suggests that most barcodes identified are likely the result of a single-cell infection, but that there are opportunities for contamination by poorly replicating genomes, remnant genomes, or cross-contamination during sequencing preparation.

Surprisingly, the light and heavy fractions were not significantly different in the distribution of number of barcodes represented, and yet both had significantly more multiple barcodes present compared to the total supernatant (*P* = 0.0021 and *P* = 0.0003, respectively [one-way ANOVA]) (Fig. 4H). The light fraction had exactly 50% single-barcode- and 50% multiple-barcode-containing plaques, whereas the heavy fraction resulted in slightly more multiple-barcode-containing plaques at 62.5% (Fig. 4G). Comparing the light fraction with the total fraction, these data suggest that additional aggregates are forming as a result of the fractionation process. This is consistent with the slight (though nonsignificant) increase in specific infectivity observed in Fig. 4C. The heavy fraction demonstrated a significant increase in specific infectivity and an increase in overall particle size but results in only nonsignificantly elevated levels of multiple barcodes present compared to the light fraction (Fig. 4C, D, and G to H). Together, these results suggest that the aggregates in the heavy fraction may be forming around the time of release from cells, since the data demonstrate that plaques are dominated by single barcodes despite multiple barcodes being present. In contrast, the aggregates in the light fraction are likely forming separate from the cell, resulting in more diverse mixes of barcoded genomes despite the smaller number of total genomes.

### Heavy ZIKV infectious units are likely aggregates rather than vesicle-bound collections of ZIKV virions.

**FIG 5:**
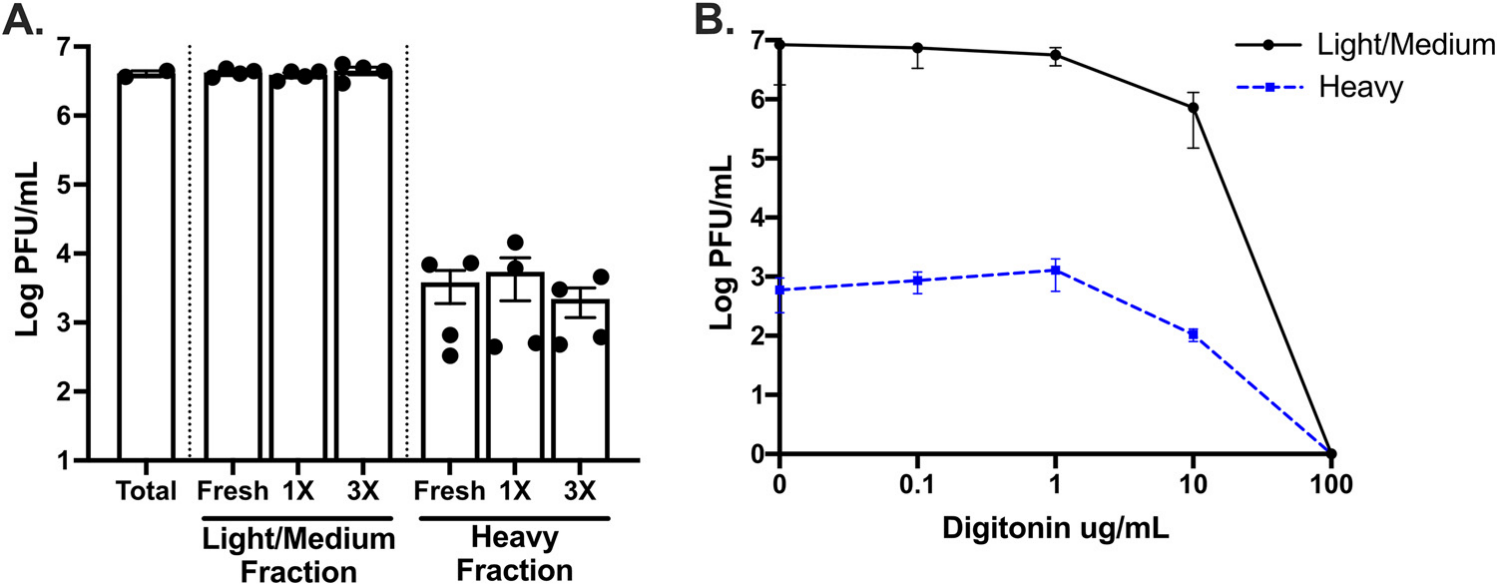
Heavy ZIKV infectious units are not sensitive to freeze-thaw cycles, and the numbers of PFU/ml do not increase after treatment with digitonin. Vero cells were infected with a ZIKV infectious clone at an MOI (PFU/cell) of 1. At 2 dpi, the supernatants were separated into two fractions: light/medium (supernatants from centrifugation at 15,000 × *g* for 8 min) and heavy (resuspended pellet after centrifugation at 15,000 × *g* for 8 min). Washes were performed to remove ≤100-nm carryover. Light/medium and heavy fractions were subjected to no, one, or three freeze-thaw cycles (A) or treated with 0, 0.1, 1, 10, or 100 μg/ml of digitonin (B). Plaque assays were performed to determine the fraction titers. Error bars represent the SEM.

Previous work demonstrates that viruses can be transmitted as vesicle-bound collective infectious units as well as through aggregates (39, 41, 43, 44, 59). Therefore, we assessed whether the ZIKV heavy fraction was vesicle-bound or unbound virion aggregates. Freeze-thaw cycles can be used to disrupt vesicles, so both the light/medium and the heavy ZIKV fractions were titrated either immediately after collection, after one freeze-thaw cycle, or after a series of three freeze-thaws. No change was seen in the titer for light/medium or heavy fractions of ZIKV for any of the freeze-thaw conditions (Fig. 5A). This suggested that the heavy fraction of ZIKV was not made up of vesicle-bound collections of viruses since no additional infectious particles were released. However, it is important to note that some extracellular vesicles have been described to be freeze-thaw resistant (60). These data also demonstrate that ZIKV virions themselves are resistant to rapid freeze-thaw cycles since there was no loss of titer from fresh preparations to those that underwent three freeze-thaw cycles (Fig. 5A).

Since some extracellular vesicles are resistant to freeze-thaw and many extracellular vesicles are enriched in cholesterol (61), we next tested whether we could disrupt vesicles while leaving virions intact using the nonionic detergent, digitonin. Digitonin is used to isolate cytosolic proteins since it disrupts the cholesterol-rich plasma membrane while leaving the membranes of organelles intact due to their far lower cholesterol content (62). Because ZIKV virions are generated on endoplasmic reticulum membranes (63), they should be more resistant to digitonin than cholesterol-rich extracellular vesicles. To determine whether ZIKV heavy fractions contained digitonin-sensitive vesicle-bound collections of particles, ZIKV light/medium or heavy particles were treated with 0.1, 1, 10, or 100 μg/ml digitonin and then titrated by plaque assay. No change in titer was observed for the light/medium or heavy fractions treated with up to 1 μg/ml digitonin. We found that 10 μg/ml digitonin resulted in an ∼1-log drop in titer for both the light/medium and the heavy fractions and continued in a dose-dependent manner for 100 μg/ml digitonin. This suggests that the heavy fraction is not made up of a significant proportion of digitonin-sensitive vesicles filled with virus, since the titer did not increase with digitonin treatment, as would be expected if vesicles were specifically disrupted by digitonin treatment. The decrease in titer observed for both ZIKV fractions treated with 10 μg/ml digitonin and undetectable titer at 100 μg/ml digitonin suggests that this is the concentration where digitonin begins to disrupt the virus envelope, inactivating virus (Fig. 5B). Overall, these data support the hypothesis that ZIKV infectious units are not appreciably packaged in vesicles for transport to new cells, suggesting ZIKV collective particle transmission is more likely a result of viral aggregates.

### The medium/heavy fraction of the ZIKV infectious population reforms after removal, and fewer PFU are present in the pellet at higher temperatures.

**FIG 6:**
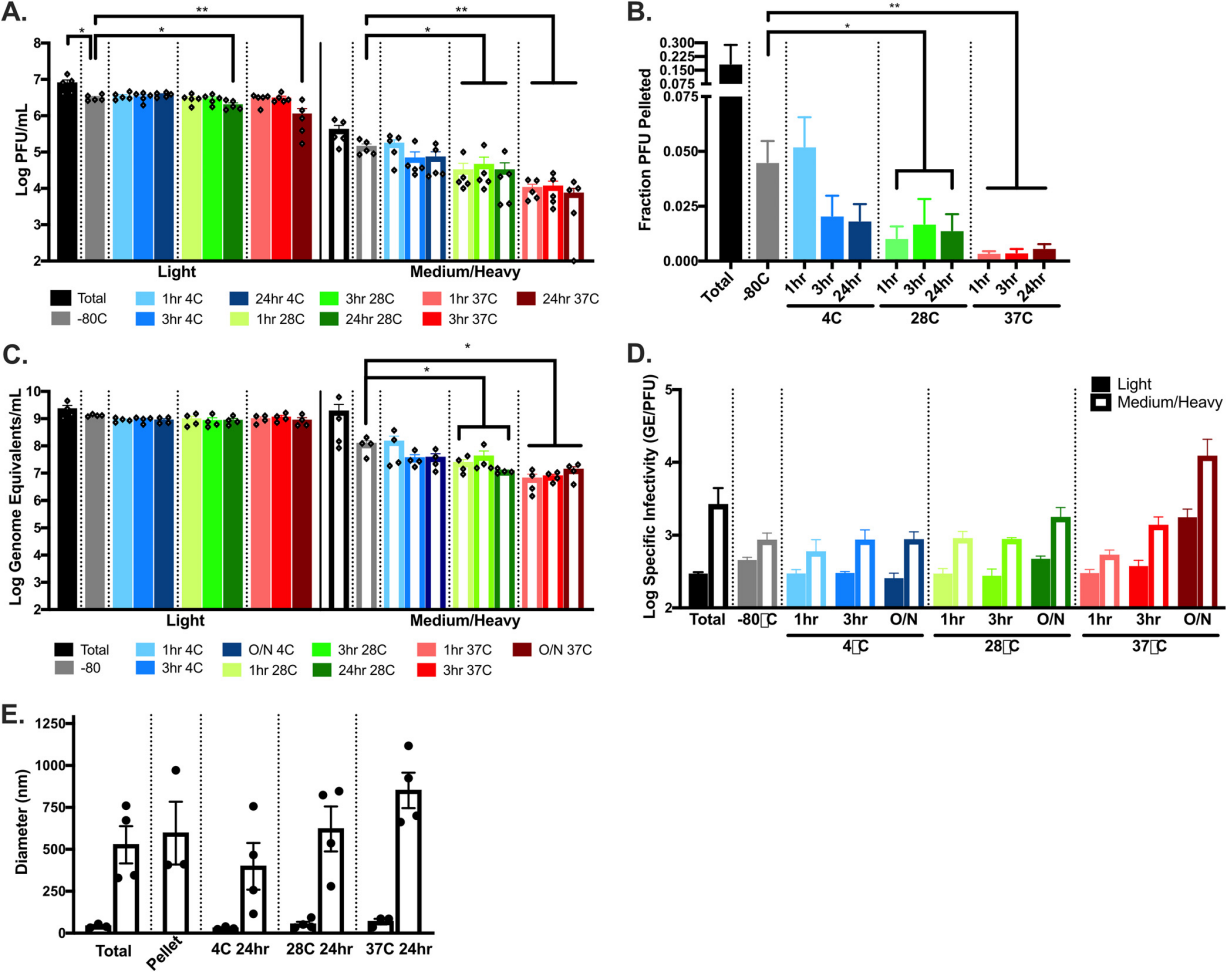
Heavy ZIKV infectious units reform after removal, and the particle diameter increases with incubation at increased temperatures. Vero cells were infected with ZIKV at MOI (PFU/cell) of 1 in 10% FBS media (A to C) or serum-free media (D). At 2 dpi, the supernatants were collected and clarified. All particles over a predicted vesicle size of 100 nm were removed by centrifugation at 21,000 × *g* for 24 min. Supernatants incubated at 4, 28, or 37°C for 1, 3, or 24 h. Immediately after the incubations, the samples were again separated into supernatant (predicted <100 nm) and pellet (predicted >100 nm) fractions, and plaque assays performed to determine the titers (A) and the fractions of PFU pelleted (B). (C) RT-qPCR from identical samples was performed to determine the genome equivalents (GE) used to assess the specific infectivity (GE/PFU). (D) Total or fractionated supernatants were similarly prepared for DLS, followed by fixation in 2.5% glutaraldehyde and dilution in PBS. Samples were read on a Zetasizer Nano ZS. Error bars represent the SEM; comparisons were determined using an unpaired *t* test (*, *P* < 0.05; **, *P* < 0.005).

Having investigated the ZIKV heavy fraction, we next sought to understand whether infectious units in the light fraction can aggregate in supernatants, separate from cellular interactions, or if medium/heavy ZIKV infectious units are generated exclusively through interactions with intact cells. To test whether ZIKV medium/heavy infectious units can reform in standard media conditions after release from cells, light fractions were generated as before. An aliquot was saved prior to initial fractionation as an input control (total). The light ZIKV infectious unit fractions were stored at −80°C, and then individual tubes were removed for incubation at 4, 28, or 37°C for 1, 3, or 24 h. Immediately after the incubations, samples (along with a light fraction sample from −80°C and the total sample) were again separated by centrifugation into light or medium/heavy fractions and then titrated by plaque assay. Surprisingly, a PFU positive pellet fraction (here, medium/heavy combined infectious particles) was again present for all samples, including those directly removed from −80°C (Fig. 6A), suggesting that aggregates are being formed during fractionation (similar to the sequencing data in Fig. 6G and H), during other processes, or that not all aggregates were removed from the supernatant. The supernatant fraction titers were relatively stable (on the logarithmic scale) across temperatures and incubation times, with only small decreases in titer for samples stored 24 h at 28 or 37°C (unpaired *t* test, *P* = 0.0160 and *P* = 0.0016, respectively) (Fig. 6A). The light fraction samples held at 37°C for 24 h retained 35.8% of infectious virus. In contrast, pellet titers dropped significantly with incubation at all temperatures except 4°C, and decreases in titer were larger with increased temperatures, resulting in decreased fractions pelleted (unpaired *t* test) (Fig. 6A and B). The medium/heavy fraction samples retained only 5.2% infectivity. These percent losses in infectivity are similar to the literature, where 10 to 15% of infectivity remained after incubation at 37°C for 24 h (64). The newly formed fraction of ZIKV pelleted PFU (−80°C) was 4-fold lower than the pelleted ZIKV population from the total (Fig. 6B), suggesting that there may be heavy particles in the original sample that are not reformed after they are removed. Overall, decreased fractions of PFU pelleted after incubation at higher temperatures could indicate that viruses—particularly those prone to being in the heavy fraction—are being degraded, that viruses more likely to be found in the heavy fraction are further aggregating at higher temperatures, or some combination of the two. To further investigate this, samples used for titration were also assayed for RNA genomes present.

### Incubation at 37°C potentially increases aggregation of ZIKV genomes.

The same samples that were titrated by plaque assay were also used to determine the GE by RT-qPCR. GE were stable for supernatants across temperatures and incubation times (Fig. 6C), suggesting that viral genomes within the light fraction are not degraded during incubation at 4, 28, or 37°C for up to 24 h. Significant decreases in GE were observed for pelleted samples incubated at 28 and 37°C (Fig. 6C), similar to the decreases in titers (Fig. 6A) (unpaired *t* test), suggesting that this population is losing some infectious viral genomes to degradation at higher temperatures, as would be expected. Interestingly, degradation seems to be skewed to the heavier populations, with no GE loss in the light fraction. To investigate whether viruses were aggregating, as well as being degraded, changes in specific infectivity (GE/PFU) were assessed across samples to determine whether temperature affects the number of genomes present per plaque formed. Pellets had more GE present per PFU compared to supernatant samples for all conditions (Fig. 6D). This is consistent with the hypothesis that the pelleted fraction consists of aggregated viruses. Samples incubated at 37°C for 24 h resulted in a 3.7-fold increase in GE per PFU in the supernatant sample and a 14.2-fold increase in the pellet (Fig. 6D), suggesting that virus particles are aggregating when incubated at 37°C. Combined, these data not only suggest that incubation of ZIKV results in degradation of infectious genomes, which increases with increased temperature, but also suggest that aggregation may be induced by incubation at higher physiologically relevant temperatures. However, these data are not strong enough to specifically conclude that aggregation, rather than only inactivation, is occurring.

To further investigate whether ZIKV particles aggregate during incubation at 4, 28, or 37°C, particle/aggregate diameters present in ZIKV-infected samples were measured by DLS. As described for light, medium, and heavy particles, Vero cells were infected with ZIKV at an MOI of 1 in serum-free media and supernatants collected at 2 dpi. ZIKV supernatants were centrifuged to remove cell debris and dust (a sample was saved as total), then fractionated into light and medium/heavy fractions. Light fractions were incubated at 4, 28, or 37°C for 24 h. Samples were fixed in 2.5% glutaraldehyde and then diluted in PBS for DLS analysis. Particle size increased with incubation at increased temperatures with light fractions incubated at 4°C demonstrating large particles with average maximum diameters of 399 nm, at 28°C of 622 nm, and at 37°C of 851 nm (Fig. 6E). Together, these data again suggest that free virions are able to aggregate and do so more readily at higher temperatures, but the findings are not conclusive.

### Replication kinetics between total, light, and heavy fractions.

**FIG 7:**
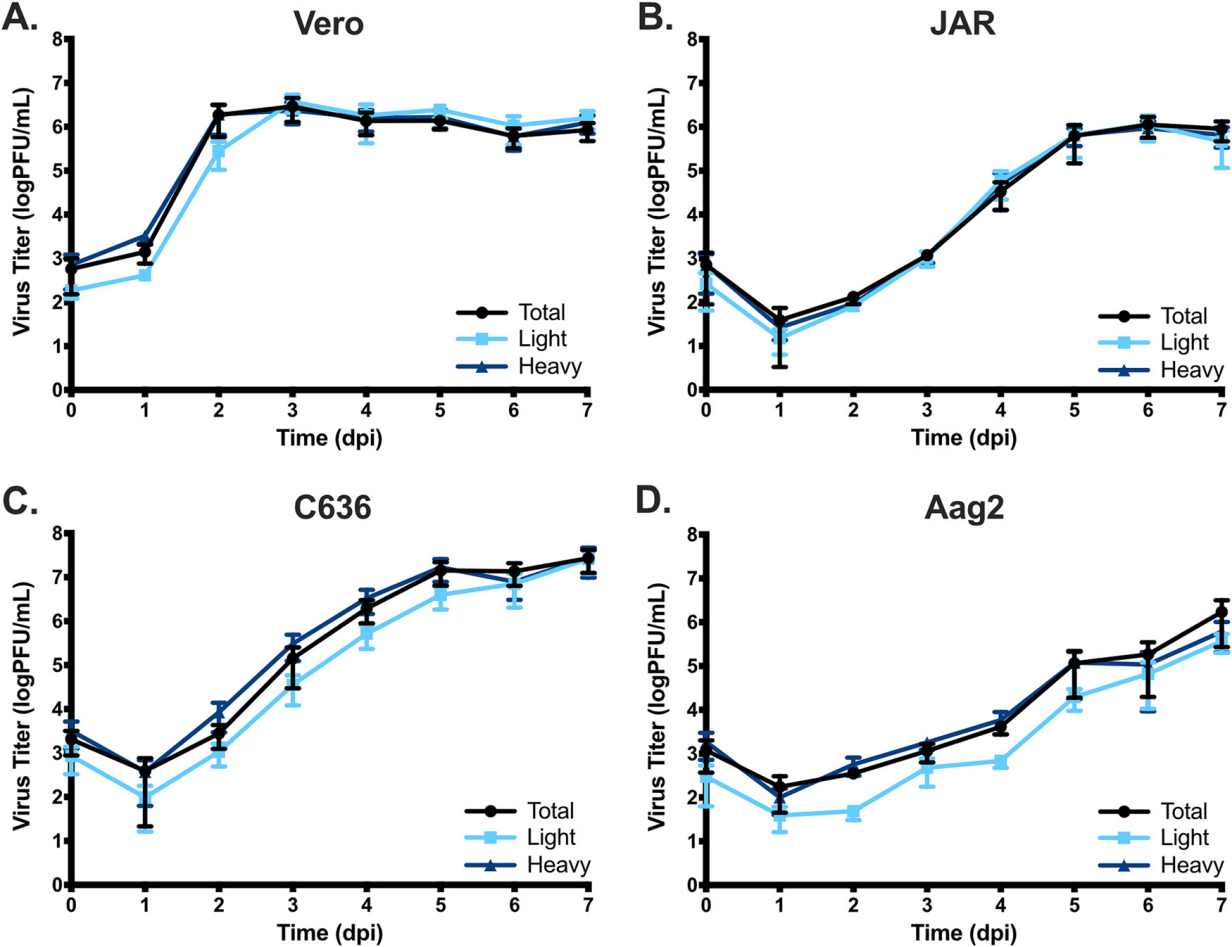
Replication kinetics are similar between weight separated groups of ZIKV infectious units in different cell types. Vero (A), JAR (B), C636 (C), or Aag2 (D) cells were infected with the titer equilibrized total, using only light or only heavy fractions of ZIKV at an MOI of 0.01 PFU/cell. Supernatant aliquots were taken at indicated days postinfection, and the titers were determined by plaque assay. Error bars represent the SEM (*n* = 4).

Having established that ZIKV infectious units likely exist as a mixed population of free and aggregated virus particles, we next sought to determine whether there was a difference in replication kinetics between fractionated populations of ZIKV. Vero, JAR, C636 (*A. albopictus*), or Aag2 (*A. aegypti*) cells were infected at an MOI of 0.01 PFU/ml with either complete populations of ZIKV (total), the light fraction alone, or the heavy fraction alone. Samples were taken from initial inoculum and at days 1 to 7 postinfection. Titers were determined by plaque assay on Vero cells. Overall, replication kinetics were identical between the total ZIKV samples and the heavy fraction population of ZIKV in all cell types tested (Fig. 7), demonstrating that there is no clear advantage or disadvantage in a cell culture setting of transmitting exclusively as large collectives or otherwise heavy particles versus the total collection of ZIKV infectious units. In contrast, in Vero, C636, and Aag2 cells there is a trend toward early delays in replication kinetics for the light-fraction samples, suggesting there might be early benefits to infection with collective ZIKV particles (Fig. 7A, C, and D). These delays in replication kinetics are most pronounced in mosquito cell lines (Fig. 7C and D). However, it is important to note that inoculum titers are lower for the light fraction is all cases, despite having been based on equilibrized titers. This may be representative of the light fraction aggregating after titration. Going forward, it would be interesting to see early replication time points and titration equilibrized by ZIKV protein or RNA rather than PFU.

## DISCUSSION

Arthropod-borne flaviviruses transmit cyclically between arthropod and vertebrate hosts, with interspecies transmission initiated by a small initial inoculum. These viruses must maintain adaption to a wide variety of cell types, across species that are separated by millions of years of evolution. RNA virus replication results in an average of one mutation per genome produced (21, 65)—the majority of mutations being deleterious to fitness (66–68)—and most viruses generate a significant portion of virions that are unable to infect subsequent cells (69). When considered together, other than due to the capacity of arthropods to serve as efficient transmission vehicles, it seems counterintuitive that arthropod-borne RNA viruses exist. However, not only do these pathogens exist, they thrive, as evidenced by dengue infecting millions of people every year and new arthropod-borne epidemics emerging regularly (1–3), as was the case with ZIKV in the new world (6, 7). One recent finding that may begin to elucidate the mechanisms behind the efficient transmission of arthropod-borne viruses is the discovery that virions do not always travel as individual units but instead can be delivered to subsequent cells and organisms as collectives (35–37, 39–41). Here, we present evidence that ZIKV can similarly be passaged as collective genomes.

We demonstrate that whereas most ZIKV plaques are composed of a single majority genotype, equally represented double genotype majorities are common in plaques (∼20%), and populations made up of multiple genotypes with none representing the majority also occur in ∼1% of plaques (Fig. 2). Further, when minority genotypes were also investigated, only one plaque sequenced presented with a single barcode (Fig. 3A and Table 1), suggesting that an even greater percentage of plaques are initiated by multiple unique genomes, potentially nearly all. After implementing strict cutoffs for what was included as a unique barcode introduced from the stock rather than via mutation, an average of 10 barcodes were identified per plaque, with the maximum observed being 212 (Fig. 3). Since plaques were intentionally selected to be spatially separated from other plaques, from wells with only three to 30 plaques, were analyzed 4 days after infection, and not all plaques picked resulted in sequencing results despite a clear cytopathic effect, these observed unique genomes are most likely the result of initial entry and replication within a single cell. However, there was evidence of a small number of RNA genomes (two and five) present in 2/24 control samples. Further supporting collective particle infection of single cells is the fact that the number of genomes found in individual plaques varies considerably. If we were sampling a lawn of virus where only a few were viable but others coated cells within a plaque, we would expect that the total number of unique genomes counted would be similar between plaques; instead, we see a large range. In addition, the number of genomes represented at 2% of the population or more within a plaque increases as the total number of genomes in a single plaque increases (Fig. 3E), suggesting that the introduction of a greater number of unique genomes increases the probability that multiple productive genomes will be present. This could result from complementation or from early innate immune evasion; both would require single cell infection (15, 70, 71). Still, we cannot formally discount the possibility that the observed numbers of unique genomes are the result of multiple cell infections in the same vicinity or persistent genomes from viruses that are not able to infect cells but remained intact near a productive infection. Future experiments can address these questions in greater detail.

We further demonstrate that ZIKV infectious units are not present exclusively as free individual virions through fractionation by low-speed centrifugation and particle diameter measurement by dynamic light scattering. ZIKV free particles have a diameter of ∼50 nm and should not be present appreciably in pellets spun down at the speeds a table-top centrifuge is capable of (maximum, <21,000 × *g*). However, we found that infectious ZIKV can be isolated in pellets typical of those used to isolate extracellular vesicles with diameters of >200 nm (Fig. 4A to C) (39, 44). This heavy fraction contains ∼1% of the total ZIKV PFU but ∼6% of the total GE, resulting in a ∼2-log higher specific infectivity for the heavy fraction compared to the total ZIKV population (Fig. 4A to C). Together, these findings are highly suggestive of collective viral transmission of ZIKV. Further this heavy fraction is made up of exclusively large particles (averaging ∼600 nm in diameter), with no 50-nm particles observed, demonstrating that free ZIKV particles are not detectable in this fraction, despite infectious units being present (Fig. 4D). However, since DLS measures diameter regardless of composition, it remains possible that the infectious units in the ZIKV heavy fraction could be bound to other nonviral ligands, increasing the diameter of an infectious unit without increasing the genomes per particle. In the case of ZIKV in FBS free media this seems unlikely, but other insect viruses do transmit via occlusion bodies and would measure larger than an individual virion without increasing the number of virions in an infectious unit such as entomopoxviruses and cytoplasmic polyhedroviruses (70). Similarly, baculoviruses transmit between hosts by occlusion bodies (OBs) and do package multiple virions in an occlusion body. Again, this seems unlikely for ZIKV in our system since OBs need to be broken down to be infectious, while the ZIKV heavy fraction is infectious upon incubation with cells in standard media. Instead, it seems most likely that ZIKV infectious units investigated here are a mix of free, minimally aggregated, and either highly aggregated or packaged virions.

Collective transmission of viral genomes has been identified in multiple systems recently and has been shown to occur by a variety of methods. Mechanisms for collective transmission of viral genomes occurs by polyploidy, direct cell-to-cell spread, accumulation of viruses on a cell surface (producing cell, distributive cell, or bacterial cell), extracellular vesicles, or viral aggregates (15, 70, 72). The ZIKV heavy fraction investigated here is of the size observed in other systems for extracellular vesicles. However, the larger and heavier ZIKV infectious units investigated in this study are suggestive of aggregates rather than vesicle-bound packages of viruses. Collective ZIKV infectious units were resistant to both freeze-thaw as well as treatment with digitonin, with no resulting change in titer compared to smaller/lighter infectious units (Fig. 5). In addition, specific infectivity and particle diameters both increased toward sizes observed for the heavy fraction generated directly from cells after incubation of light particles at increased temperatures (Fig. 6D and E). VSV virion similarly aggregate when incubated at 37°C (35), whereas extracellular vesicles would not be restored after removal in the absence of cellular replication. In addition, the high average barcode diversity observed per plaque (Fig. 3D), combined with persistence of multi-barcode majorities after infection in mosquito tissues (Fig. 2C), suggests aggregation occurring after egress from multiple parental cells. These observations are also consistent with transmission electron microscopy studies of ZIKV where both free and closely associated ZIKV virions are observed, but not extracellular vesicles (73). In addition, ZIKV has been observed to exist in highly organized sheets within infected cells, demonstrating the ability of ZIKV to form consistent, organized interactions with itself (73). In contrast, enteroviruses were shown to transmit by extracellular vesicles but in the majority of cases viruses within vesicles seem to be generated from identical parental genomes even when generated from coinfected cells (44). Instead, if aggregates form outside the cell they may have a greater opportunity for maintaining and accumulating diversity, which could be of particular importance for mosquito-borne viruses that need to transmit between distantly related hosts and escape siRNA targeting. However, sequencing data comparing the heavy fraction with the light fraction and the total supernatant may suggest that the largest ZIKV aggregates are formed in or near generating cells since the number of barcodes present (while more numerous than the total fraction) is similar to the light fraction, this despite a substantially elevated genome/PFU ratio in the heavy fraction. This could result from aggregates that are resulting from genomes biased toward a single genotype, as is seen throughout the sequencing data for individual plaques. In contrast, in the light fraction, since fractionation results in aggregation from previously free virions (potentially a result of centrifugation), small aggregates could contain more diversity relative to the aggregates formed in or near generating cells.

Collective transmission of viruses in the absence of mutations, selective factors, and defective viral genomes would be detrimental to viral propagation since it would lower the effective number of virions able to start new cell infections. However, RNA viruses exist in environments where mutations and deletions are continuously generated and host immune systems are working/adapting tirelessly to eradicate viruses. The benefits of viral collective transmission are just beginning to be identified and elucidated. Rotavirus was shown to be more infectious when administered as vesicle-bound collective particles than as free particles when fed to mice at identical viral protein concentrations (39). Similarly, vesicular stomatitis virus (VSV) is more infectious when driven to aggregation by incubation in saliva (71). Interestingly, VSV-mutagenized populations lost this benefit of aggregation, suggesting that genetic complementation was not likely to be the driver of increased fitness in this system. Instead, aggregation was most beneficial when innate immune functions were most intact (71), suggesting that the delivery of collective infectious units was more likely to be due to some other benefit of multiple genomes entering a cell, such as increased initial protein generation resulting in increased initial RNA genome production. For coxsackievirus packaged in vesicles, collectively packaged viruses were found to be generated most commonly from identical parental genomes, further expanding the idea that collective transmission of viruses may be beneficial through mechanisms beyond genetic complementation (44). However, in flaviviruses (and many others), particularly at high MOIs, defective viral genomes are known to exist and be carried along with infectious viruses even in natural settings, demonstrating that complementation does occur (69, 74). Our data suggest that there are no strong benefits or detriments to infection with highly collective versus small aggregate viral particles during replication in cell culture (Fig. 7) but that early replication should be considered more closely. It will be interesting to see in future experiments if there are benefits to ZIKV at early time points, or in more immunologically intact systems, to infection by individual versus collective particles. Similarly, it is possible that a virus stock with more inherent diversity, such as a natural isolate, would demonstrate differential replication kinetics particularly during a natural transmission cycle.

This study is the first to investigate whether flavivirus infections, ZIKV infections in particular, are generated from single or multiple parental genomes. Overall, productive infections initiated by individual genotypes seems to be a rare occurrence, despite free particles likely being available in supernatants. However, single genotypes end up dominating replication in plaques. ZIKV virions are capable of aggregating outside cells, but the mechanisms of aggregation need to be further explored. Many questions remain regarding the contribution of these aggregates to ZIKV biology. Do aggregates contribute to efficient infection of cells? What are the mechanisms of competition and complementation of different genotypes within a single cell infection? Are aggregates formed more readily in certain systems/tissues? What is the relevance of aggregates versus free virions to infection in whole organisms? The present study serves as a first step toward answering these essential evolutionary questions in flavivirus biology.

## MATERIALS AND METHODS

### Cell culture.

The Vero (African green monkey kidney; ATCC CCL-81) cell line was maintained in Dulbecco modified Eagle medium (DMEM; Corning), supplemented with 10% fetal bovine serum (FBS; Peak Serum), 1% penicillin and streptomycin (Pen-Strep; Corning), HEPES (Gibco), and 0.1% amphotericin B (Geminin Bio-Products). The JAR (Human Placental; ATCC HTB-144) cell line was maintained in RPMI medium (Corning) supplemented with 10% FBS and 1% Pen-Strep. Vertebrate cell lines were maintained at 37°C with 5% CO_2_ and propagated to new flasks using 0.05% trypsin (GE Healthcare Life Sciences). The C636 (*A. albopictus*; ATCC CRL-1660) cell line was maintained in MEM (GE Healthcare Life Sciences) with 10% FBS and 1% Pen-Strep. The Aag2 (*A. aegypti*; RRID:CVCL_Z617) cell line was maintained in Schneider’s insect medium with 10% FBS and 1% Pen-Strep. Both insect cell lines were maintained at 28°C, C636 with 5% CO_2_, Aag2 in sealed flasks, and propagated to new flasks by cell scraping.

### Mosquito colonies and maintenance.

Aedes aegypti mosquito colonies originally established from wild populations in Poza Rica, Mexico (75), were maintained at a constant temperature of 28°C and a relative humidity of 70%. Light was also controlled with a 12:12-light/dark diurnal cycle. Adult mosquitoes were provided sucrose and water *ad libitum*. Mosquito infection experiments with ZIKV were performed exclusively on female mosquitoes and under BSL3 conditions.

### ZIKV strain: cloning, recovery, and verification of bar-ZIKV virus.

All experiments use the PRVABC59 ZIKV (GenBank accession number KU501215) (76) infectious clone (77) at passage 1 or bar-ZIKV at passage 0. To generate the second-generation bar-ZIKV virus, barcode 1 from Weger-Lucarelli et al. (52) with eight degenerate nucleotides within the NS2a (Fig. 1A) RCA amplified plasmid was generated as described except the barcoded fragment was amplified in four separate reactions prior to plasmid assembly by NEB Hifi Assembly to enhance diversity. In addition, digested RCA reactions were transfected into 293T cells instead of Vero cells to enhance transfection efficiency and to prevent second cycle infection since 293T cells are permissive but not susceptible to ZIKV (78). Virus was collected at 3 dpi, and then the titer was measured by tragacanth gum overlay plaque assay on Vero cell monolayers as previously described (79), except monolayers were infected with serial 10-fold dilutions of virus stock, and plaques were counted and multiplied by dilution factor to determine viral titer/ml. Viral sequence and diversity at the barcode degenerate sites were initially confirmed by NGS (Fig. 1) and Sanger sequencing (Fig. 2A).

### Generation of mosquito samples for plaque purification.

To generate legs and wings samples, *A. aegypti* mosquitoes were injected with a 138-nl bar-ZIKV stock (∼150 PFU/mosquito) using a Nanoject II (Drummond Scientific); at 4, 7, or 11 days postinjection, mosquitos were cold anesthetized and kept on ice, while legs and wings were removed. Legs and wings were stored in 500 μl of mosquito diluent as previously described (77); however, tissues were not homogenized but instead allowed to perfuse so as not to disrupt any ZIKV collective particles (aggregates or vesicles).

### Plaque purification and Sanger sequencing of bar-ZIKV-infected cells.

Vero cell monolayers, in 6-well plates at 70 to 90% confluence, were infected with bar-ZIKV P0 stock virus or samples from *A. aegypti* mosquito legs and wings in 10-fold serial dilutions. Cells were overlaid with 0.6% agar in Eagle minimal essential medium (EMEM) and allowed to replicate for 3 days, at which time a secondary agar-EMEM overlay was added with 0.015% Neutral Red solution (Sigma-Aldrich). Plaques were picked by pipette 24 h after the addition of Neutral Red solution from wells containing 1 to 30 plaques. Only plaques that were spatially separated from all other plaques were selected to minimize potential false multiple genomes. Plaque plugs were pipetted into 150 μl of 10% FBS/DMEM and allowed to incubate for 1 h at 37°C to release virus from the agar. RNA was extracted from isolated plaques using a Mag-Bind viral DNA/RNA 96 kit (Omega Bio-Tek) on a KingFisher Flex magnetic particle processor (Thermo Fisher) as previously described (80). cDNA was generated using SuperScript III reverse transcriptase enzyme (Invitrogen) with random hexamers, and then an amplicon, including the barcoded region of ZIKV, was amplified using EasyA High-Fidelity PCR cloning enzyme and primers (forward, 5′-GCCCAGGAAAGAACCAGAAA-3′; reverse, 5′-CTTCACACTGCCTTTTCCCT-3′). PCR products were purified using NucleoSpin Gel and PCR Clean-up kit (Macherey-Nagel) according to the manufacturer’s protocol. Purified PCR products were sent for Sanger sequencing across the barcode (Genewiz). Alignments were performed using Geneious software.

### Next-generation sequencing library preparation for deep sequencing of barcoded genomes.

Viral RNA from plaque picks used for Sanger sequencing above, was prepared for next-generation sequencing using PrimerID amplicon sequencing, which allows the validation of template sampling depth and reduction of the error rate when assessing genetic diversity in the barcode region, as previously described (54). Briefly, cDNA of the barcoded region was generated using SuperScript III reverse transcriptase enzyme (Invitrogen) with the barcode locus-specific PrimerID primer ID_ZIKV_4121_R (5′-GTCTCGTGGGCTCGGAGATGTGTATAAGAGACAGNNNNNNNNNcagtCTTCACACTGCCTTTTCCCT-3′), followed by RNase H treatment and purification using Ampure XP beads (Beckman Coulter). PCR amplification was performed using KAPA HiFi HotStart ReadyMix (Roche). The first-round PCR forward primer ID_ZIKV_3936_F (5′-TCGTCGGCAGCGTCAGATGTGTATAAGAGACAGNNNNGGTTTTGCTTTGGCCTGGTT-3′) was used for the ZIKV-barcoded region, as well as the reverse primer ID_R (5′-GTCTCGTGGGCTCGGAGATGTGTAT-3′). First-round PCR products were purified using Ampure XP bead (Beckman Coulter). The second-round PCR forward primer (5′-CAAGCAGAAGACGGCATACGAGAT[i7]GTCTCGTGGGCTCGG-3′) and reverse primer (5′-AATGATACGGCGACCACCGAGATCTACAC[i5]TCGTCGGCAGCGTC-3′) were used to incorporate unique Nextera XT i7 and i5 indexes for each sample processed for complete dual indexed libraries. Final libraries were pooled and analyzed for size distribution using the Agilent high-sensitivity D1000 Screen Tape on the Agilent Tapestation 2200. The final quantification was performed using the NEBNext Library Quant kit for Illumina (NEB) according to the manufacturer’s protocol. Libraries were then sequenced on an Illumina HiSeq 4000 using 2 × 150 paired-end reads (Genewiz) or an Illumina MiSeq v2 using 2 × 250 paired-end reads (for libraries generated from plaque picks after fractionation).

### Deep-sequencing analysis.

Next-generation sequencing data were processed to characterize the barcodes and their frequencies. We developed a workflow called “Primer_ID_Barcoded Analysis” (https://bitbucket.org/murrieta/primer-id-with-barcoded-virus/src/master/) that makes use of the template consensus sequences (TCS) pipeline (54), the bbmap suite of tools (https://sourceforge.net/projects/bbmap/), and custom perl scripts to process and analyze PrimerID-generated barcoded virus sequence data. Briefly, paired-end reads were processed through the TCS pipeline to generate consensus sequences for each template sequenced, and bbduk was used on these paired-end consensus sequences to find reads containing 20 nucleotides upstream and downstream of the barcode regions. bbmap was used to map the remaining reads to the Zika reference (GenBank accession no. KU501215) barcode region (nucleotides 3623 to 4338), which were then oriented and trimmed using bbduk so that only the 24-bp barcode remained. Custom perl scripts were used to identify and count unique barcodes.

### Fractionation of ZIKV infectious population by low-speed centrifugation.

Vero cells were infected with a ZIKV infectious clone at an MOI (PFU/cell) of 1 in 10% FBS media for titration and RNA quantification or in serum-free media for DLS analysis. At 2 dpi in Vero cells (5 dpi for JAR), supernatants were collected and centrifuged at 500 × *g* for 6 min to remove cell debris and dust. Clarified supernatants were then separated into three fractions: light (predicted <100-nm vesicle size; supernatants from centrifugation at 21,000 × *g* for 24 min), medium (predicted 100- to 200-nm vesicle size; pellet from 21,000 × *g* for 24 min resuspended, and then the supernatant recovered after centrifugation at 15,000 × *g* for 8 min), and heavy (resuspended pellet after centrifugation at 15,000 × *g* for 8 min). Four washes were performed between changes in centrifugation speed to the remove 50- or 100-nm carryover, respectively. All centrifugation steps were performed at 4°C. Plaque assays were performed to determine fraction titers immediately after separation. Centrifugation speeds were determined using the Laboratory of Molecular Human Genetics, Research Institute of Physical-Chemical Medicine online calculator (http://vesicles.niifhm.ru/index.php?do=1).

### Determination of genome equivalents and specific infectivity by qRT-PCR.

An RNA standard was generated using ZIKV infectious clone plasmids to generate a full-length genomic RNA, as described previously (77). Dilutions of the standard curve were made from 10^2^ to 10^7^ GE for use in assay as needed. Supernatant samples from fractionated ZIKV supernatants (above) and temperature studies (below) were used to extract RNA using the Mag-Bind viral DNA/RNA 96 kit (Omega Bio-Tek) on a KingFisher Flex magnetic particle processor (Thermo Fisher) as previously described (80). The primers and probes used were described previously: briefly, we used probe 56-FAM/AGCCTACCT/ZEN/TGACAAGCAGTCAGACACTCAA/3IABkFQ, forward primer 5′-CCGCTGCCCAACACAAG-3′, and reverse primer 5′-CCACTAACGTTCTTTTGCAGACAT-3′ (81). An Express One-Step SuperScript qRT-PCR kit (Thermo Fisher) was used with a final volume for the reactions of 20 μl: 50 nM probe, 1 μM concentrations o0f each primer, 5 μl of sample RNA, 10 μl of 2× Supermix, 1 μl of reverse transcriptase, and 1.5 μl of H_2_O. Samples were plated in duplicate and run under the following conditions: 50°C for 15 min, 95°C for 5 min, 95°C for 15 s, and 60°C for 1 min, with the last two steps repeated 40×. The standard curve was graphed, and the genomes per ml were determined. Identical samples were used for determination of PFU/ml and paired to GE/ml to determine specific infectivity, and the ratio of GE to PFU of the supernatant was determined.

### Dynamic light scattering analysis of ZIKV-infected supernatants.

All samples analyzed by DLS were prepared in FBS-free media. Samples were fixed in 2.5% glutaraldehyde (Sigma-Aldrich) and allowed to incubate for 1 h at 37°C. After fixation, the samples were diluted 1:10 in 0.22-μm sterile-filtered phosphate-buffered saline (PBS) without calcium or magnesium (Corning) and transferred to disposable polystyrene cuvettes. Samples were read on a Malvern Zetasizer Nano ZS spectrometer at 25°C. The PBS composition was programed into Zetasizer software to determine the viscosity. The particle density was set to mimic exosomes and WNV particles with a refractive index of 1.390, and the adsorption was set at 0.001. The “Protein Analysis” setting within “Data Processing” was used. Control samples were run from uninfected cell supernatants at matched times postplating to ensure that particle sizes were a result of infection rather than a normal part of the cellular physiology. Samples were only included when the cumulant fit data were strong and the polydispersity index was not too high for a consistent reading. Inferred diameter sizes were calculated by using Zetasizer Nano ZS software, and only the three most represented particle sizes are presented in the software analysis.

### Freeze-thaw and digitonin disruption of ZIKV.

Subconfluent Vero cells were infected with ZIKV infectious clone passage 1 at an MOI of 1 PFU/cell. At 2 dpi, the supernatants were collected and centrifuged at 500 × *g* for 6 min to remove cell debris and dust. Clarified supernatants were then separated into two fractions: light/medium fractions with a predicted diameter of <200 nm (supernatants from centrifugation at 15,000 × *g* for 8 min) and heavy fractions with a predicted diameter of >200 nm (resuspended pellet after centrifugation at 15,000 × *g* for 8 min). Four successive washes, with 1 ml of 10% FBS/DMEM and centrifugation at 15,000 × *g* for 8 min, were performed to remove the ≤100-nm carryover in the heavy fraction. In freeze-thaw studies, titers for both light/medium and heavy fractions were determined by a tragacanth gum overlay plaque assay immediately (no freeze), or the fractions were frozen at −80°C for 10 min, followed by thawing at 37°C for 5 min one or three times, and then the titers were determined by plaque assay as described above. Digitonin (Sigma-Aldrich) was prepared in water to 1 mg/ml by heating to 98°C for 1 h. For the digitonin assay, both light/medium and heavy fractions were treated with 0, 0.1, 1, 10, or 100 μg/ml digitonin in digitonin buffer (150 mM NaCl, 50 mM HEPES [pH 7.4], 0.22-μm filtered) (62). ZIKV fractions were incubated with digitonin for 10 min on ice before titration. Again, tragacanth gum overlay plaque assays were performed to determine the fraction titers.

### Temperature incubation studies.

Subconfluent Vero cells were infected with a ZIKV infectious clone at an MOI (PFU/cell) of 1 in 10% FBS/DMEM or serum-free DMEM (for DLS analysis). At 2 dpi, the supernatants were collected and centrifuged at 500 × *g* for 6 min to remove cell debris and dust. Clarified supernatants were centrifuged at 21,000 × *g* for 24 min to remove all particles over a predicted vesicle size of 100 nm. Supernatants were stored at −80°C then incubated at 4, 28, or 37°C for 1, 3, or 24 h. Immediately after the incubations, the samples were again centrifuged at 21,000 × *g* for 24 min, and the supernatants were removed to separate the fractions with predicted particle sizes of <100 nm. The pellets were washed four times in DMEM at centrifugation speeds of 21,000 × *g* for 24 min to remove small particle carryover and then resuspended to generate the >100-nm fractions.

### Virus replication kinetics assays.

Subconfluent Vero cell monolayers in triplicate were infected at an MOI of 0.01 PFU/cell. Virus was allowed to adsorb for 30 min when inocula were removed, and the cells were washed 2× with PBS, followed by the addition of prewarmed media with reduced (2 or 1%) FBS. Samples were taken at various time points postinfection. Titers were determined by plaque assay as described above.

### Statistical analysis.

Statistics were applied as described in the figure legends by using Prism 6 software (GraphPad, La Jolla, CA). The number of replicates performed for each experiment is either listed in each figure legend or represented individually in the graphs. Finally, some data were normalized to the controls; GraphPad Prism 6 software also performed this analysis.

**TABLE 1:**
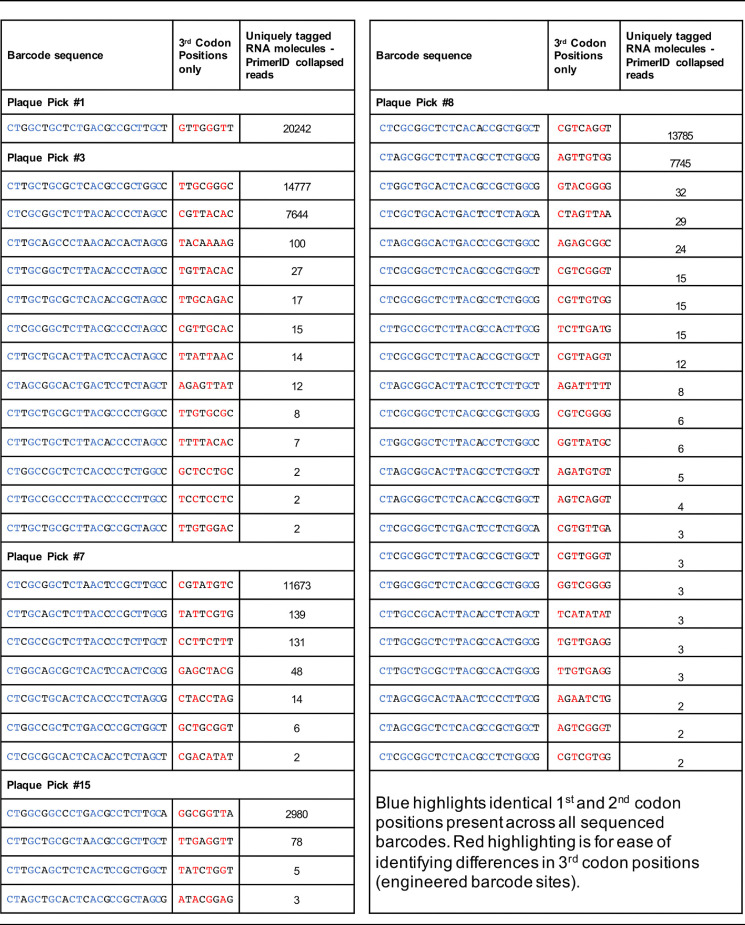
Representative barcode data^*a*^

a RNA from ZIKV barcode-infected plaques was transcribed to cDNA using PrimerID tags to bioinformatically identify individual RNA molecules. Illumina sequencing was performed across the barcoded region. Complete barcode data, with number of RNA molecules detected is presented for a subset of sequenced plaques. Blue highlights identical first and second codon positions present across all sequenced barcodes. Red highlighting is for ease of identifying differences in third codon positions (engineered barcode sites).
